# Mitochondrial Peroxiredoxin 3 Regulates Sensory Cell Survival in the Cochlea

**DOI:** 10.1371/journal.pone.0061999

**Published:** 2013-04-23

**Authors:** Fu-Quan Chen, Hong-Wei Zheng, Jochen Schacht, Su-Hua Sha

**Affiliations:** 1 Department of Pathology and Laboratory Medicine, Medical University of South Carolina, Charleston, South Carolina, United States of America; 2 Department of Otolaryngology, Kresge Hearing Research Institute, University of Michigan, Ann Arbor, Michigan, United States of America; Oregon Health & Science University, United States of America

## Abstract

This study delineates the role of peroxiredoxin 3 (Prx3) in hair cell death induced by several etiologies of acquired hearing loss (noise trauma, aminoglycoside treatment, age). *In vivo*, Prx3 transiently increased in mouse cochlear hair cells after traumatic noise exposure, kanamycin treatment, or with progressing age before any cell loss occurred; when Prx3 declined, hair cell loss began. Maintenance of high Prx3 levels via treatment with the radical scavenger 2,3-dihydroxybenzoate prevented kanamycin-induced hair cell death. Conversely, reducing Prx3 levels with Prx3 siRNA increased the severity of noise-induced trauma. In mouse organ of Corti explants, reactive oxygen species and levels of Prx3 mRNA and protein increased concomitantly at early times of drug challenge. When Prx3 levels declined after prolonged treatment, hair cells began to die. The radical scavenger *p*-phenylenediamine maintained Prx3 levels and attenuated gentamicin-induced hair cell death. Our results suggest that Prx3 is up-regulated in response to oxidative stress and that maintenance of Prx3 levels in hair cells is a critical factor in their susceptibility to acquired hearing loss.

## Introduction

Oxidative stress is a common contributor to disease and tissue injury, making the maintenance of intracellular redox homeostasis a decisive determinant for cell death or survival. Peroxiredoxins are components of a ubiquitous thioredoxin-dependent antioxidant defense system that catalyzes the inactivation of reactive oxygen species (ROS) or their progenitors, including hydrogen peroxide (H_2_O_2_) and peroxynitrite (ONOO^−^), as well as the reduction of protein sulfhydryl groups [1], [2]. The six known isoforms of the family of mammalian peroxiredoxins exhibit cell-specific distribution patterns and are localized to different cellular compartments. Of these, Prx3 is a mitochondrion-specific enzyme required to maintain normal mitochondrial metabolism and integrity [3]. Underscoring its role as an essential antioxidant, overexpression of Prx3 protects the rat hippocampus from excitotoxic injury and the mouse myocardium from infarction [4], [5]. Conversely, depletion of Prx3 results in increased intracellular levels of H_2_O_2_ and sensitizes cells to apoptotic signaling [6], [7]. Prx3 knock-out mice also had high intracellular ROS levels and increased sensitivity to tissue damage in lipopolysaccharide-induced lung inflammation [8].

Antioxidant status has emerged as a determining factor for cell death or survival in the inner ear in response to three major contributors to acquired hearing loss: noise trauma, ototoxic medications (aminoglycoside antibiotics, cisplatin), and the aging process. Indicators of oxidative stress have been demonstrated in the inner ear for all three traumata [9]–[11] and, underscoring a crucial role in cell fate, maintenance of cochlear redox homeostasis has the potential to improve hair cell survival [12]–[14]. Hair cells, the sensory cells of the organ of Corti responsible for the transduction of acoustic input into nerve impulses, are primary targets of these insults. Damage to or loss of hair cells leads to permanent hearing loss, as mammalian hair cells lack the ability to regenerate.

The details of interactions between the various insults and endogenous antioxidant pathways remain to be established. Mitochondria are a major source of cellular ROS and there is evidence to suggest that noise-, aminoglycoside-, and aging-induced oxidative stress might originate in the mitochondria [15]–[17]. Over-production of ROS induces mitochondrial swelling, degeneration, and the release of cytochrome c, as well as activation of caspase-3-dependent cell death pathways [9], [18], [19]. In the current study, we hypothesize that Prx3 levels are critical in hair cell susceptibility to trauma via regulation of mitochondrial redox balance. The animal of choice is the CBA/J mouse for which *in-vivo* models for all three pathologies have been well established [20]–[22]. In addition, we address the potential regulation of Prx3 mRNA levels in explants of the mouse organ of Corti [15], [23].

## Materials and Methods

### Materials

The primary antibodies used in this study are polyclonal rabbit anti-Prx3 for immunohistochemistry (ProteinTech Group Inc, Chicago, IL, # 10664-1-AP), monoclonal mouse anti-Prx3 for Western blotting (Abcam, Cambridge, UK, # ab16751), polyclonal rabbit anti-GAPDH (Millipore, Billerica, MA, # ABS16), and polyclonal rabbit anti-Myosin VII (Proteus Bioscience, Ramona, CA, # 25-6790). Prx3 siRNA (# MSS202040_a1N), goat anti-rabbit secondary antibody conjugated to Alexa Flour® 488, Alexa Fluor® 594, Hoechst 33342, 3′-(*p*-aminophenyl) fluorescein (APF), MitoTracker® Red CMXRos, BenchMark Prestained Protein Ladders, SuperScript^III^ First-Strand Synthesis system for RT-PCR, Insulin-Transferrin-Selenium-A Supplement, and MasterMix were purchased from Invitrogen Life Technologies (Carlsbad, CA). Secondary antibodies for Western blotting were obtained from Jackson ImmunoResearch Laboratories (West Grove, PA). RIPA buffer was purchased from Cell Signaling Technology, Inc. (Danvers, MA), Complete Mini EDTA-free protease inhibitor cocktail tablets from Roche Diagnostic GmbH (Mannheim, Germany), RNeasy Micro Kit from Qiagen (Valencia, CA), and Enhanced Chemiluminescence Plus from Amersham Pharmacia Biotech (Piscataway, NJ). RNA*later*® was purchased from Ambion (Austin, TX), TaqMan primer and probes from Applied Biosystems (Foster City, CA), ABC solution from Vector Laboratories Inc. (Burlingame, CA), and protein assay reagents from Bio-Rad (Hercules, CA). Kanamycin A sulfate was purchased from USB Corporation (Cleveland, OH). Gentamicin and all other reagents were from Sigma-Aldrich Co. (St. Louis, MO).

### In-vivo Experiments in Mice

#### Animals

For use in all *in-vivo* experiments, male CBA/J mice were purchased from Harlan Sprague Dawley (Indianapolis, IN) at 12 weeks of age for noise exposure and at 4 weeks for kanamycin treatment. For the aging study, 18-month old CBA/J male mice were obtained from the National Institute on Aging. For breeding, one male and two female mice at 6–8 weeks of age were placed in one cage. Mice were allowed free access to water and a regular mouse diet (Purina 5025, St. Louis, MO), were kept at 22±1°C under a standard 12 h:12 h light-dark cycle, and were allowed to acclimate for one week prior to the studies. All research protocols were approved by either the University of Michigan (UM) Committee on Use and Care of Animals or by the Institutional Animal Care & Use Committee at the Medical University of South Carolina (MUSC). Animal care was either under the supervision of the Unit for Laboratory Animal Medicine at UM or under the supervision of Division of Laboratory Animal Resources at MUSC.

#### Noise exposure

Mice in individual stainless steel wire cages (approximately 9 cm × 9 cm × 9 cm) were exposed to broadband noise (BBN) with a frequency spectrum from 2–20 kHz at 106 dB SPL for 2 hours, with the exception of the Prx3 siRNA *in-vivo* studies, in which mice were exposed to 96 dB SPL. The exposure chamber was the same as we described previously [20]. Control mice were kept in silence within the same chamber for 2 hours.

#### Drug administration

Kanamycin is the aminoglycoside of choice for *in-vivo* studies in mice [22]. Animals were separated into four groups to receive 15 days of twice-daily subcutaneous injections of either: group 1: kanamycin sulfate (700 mg of kanamycin base/kg body weight) in saline, group 2: kanamycin and 2,3-dihydroxybenzoate (DHB) (300 mg DHB/kg body weight in 4% sodium bicarbonate) in separate but simultaneous injections, group 3: DHB only, or group 4: saline only.

#### Prx3 siRNA delivery to the inner ear

The methods for Prx3 siRNA delivery into the adult mouse inner ear *in viv*o were modified from a recent study [24]. Briefly, after anesthesia, a retroauricular incision was made to approach the temporal bone. The otic bulla was identified ventrally to the facial nerve and a shallow hole was made in the thin part of the otic bulla with a 30 G needle and enlarged with a dental drill to a diameter of 2 mm in order to visualize the round window. A customized sterile Micro Medical tube was inserted into the hole just above the round window to slowly deliver 10 µL (0.6 µg) of Prx3 siRNA. After the siRNA delivery, the hole was covered with surrounding muscle and glued with tissue adhesive. Finally, the skin incision was closed with tissue adhesive and the mouse was kept in the surgical position for 1 hour. Three days post-Prx3 siRNA delivery, mice were exposed to BBN.

#### Auditory Brainstem Response (ABR)

Mice were anesthetized with intra-peritoneal injections of xylazine (20 mg/kg) and ketamine (100 mg/kg). ABR measurements were performed as previously described [20]. All ABR measurements were evaluated by an expert blinded to the treatment conditions.

#### Surface preparation and DAB staining of hair cells

The methods for surface preparation and DAB staining of hair cells were followed as reported previously [20]. Images were taken with a Zeiss AxioCam MRc5 camera with Axioplan 2 imaging software under a Zeiss microscope.

#### Hair cell counts on cochlear epithelia from the adult mouse

Images from the apex through the base of the cochlear epithelium stained for myosin VII and DAB were captured using a 40× lens on the Zeiss microscope. The lengths of the cochlear epithelia were measured and recorded in millimeters. The three rows of OHCs in the 4.0–5.0 mm segments (distance from apex) of the basal region were counted. The average number of OHCs per 100 µm in the basal region was evaluated for statistical significance.

#### Immunofluorescence of Prx3 on surface preparations from adult mice

Following fixation with 4% paraformaldehyde for overnight and decalcification with 4% EDTA for 72 hours at 4°C, cochleae were dissected under a microscope by removing the softened otic capsule, stria vascularis, Reissner’s membrane and tectorial membrane. The remaining tissue, including the modiolus and cochlear sensory epithelium, was permeabilized in 3% Triton X-100 solution for 30 min at room temperature. The specimens were washed three times with PBS and blocked with 10% normal goat serum for 30 min at room temperature, followed by incubation with rabbit anti-Prx3 (1∶50) at 4°C for 72 hours. After washing three times with PBS, the secondary goat anti-rabbit antibody (Alexa Fluor 488-conjugated, 1∶200) was applied at 4°C overnight in darkness. For fluorescent visualization of hair cell structure, the specimens were incubated with Alexa Fluor 594 phalloidin (1∶100) at room temperature for 30 min. After the final wash with PBS, surface preparations were obtained by removing the modiolus. The epithelia were divided into three segments (apex, middle, and base). Specimens were mounted on slides with anti-fade mounting media and imaged on a Zeiss confocal microscope.

#### Quantification of immunofluorescence signals on surface preparations

Immuno-fluorescence of Prx3 on surface preparations was quantified from original confocal images, each taken with a 63× magnification lens under identical conditions and identical settings for laser gains and PMT gains, using ImageJ software (National Institute of Health, Bethesda, MD). The cochleae from groups to be directly compared, for example noise-exposed and unexposed groups, were fixed and immunolabeled simultaneously with identical solutions and processed in parallel. All surface preparations were counter-labeled with Alexa Fluor 594 phalloidin for visualizing hair cell structure in order to identify comparable parts of the hair cells for the capture of confocal images. The borders of individual OHCs were outlined based on the phalloidin immunolabeling. The immunofluorescence of Prx3 in hair cells was measured in the upper-basal cochlear surface preparations in 0.12-mm segments, each containing about 60 OHCs. The intensity of the background fluorescence was subtracted and the average fluorescence per cell was calculated. The relative fluorescence was quantified by normalizing the ratio of average fluorescence of the treated group to the average fluorescence of the control group. Each condition was replicated in three different animals.

### Experiments with Mouse Organotypic Culture Preparations

#### Organotypic cultures

Organ culture explants were prepared as reported previously [23]. Briefly, mice at the age of postnatal day 3 (p3) were used; cochlear epithelia were isolated. After 48 hours of incubation to allow for recovery from dissection stress, the explants received fresh medium with individual drugs or drug combinations. Gentamicin is the preferred aminoglycoside for studies in cochlear explants (Choungh et al., 2009; Chen et al., 2009; Yu et al., 2009); it was replaced by kanamycin for the *in-vivo* studies because it is not tolerated by mice (Wu et al., 2001). Final concentrations in the incubations were 200 µM gentamicin (or 500 µM sodium sulfate for controls), 60 µM 10058-F4 (Z,E)-5-(4-ethylbenzylidine)-2-thioxothiazolidin-4-one, 0.15% dimethyl sulfoxide, or 1 mM *p*-phenylenediamine (PPD).

#### Western blotting

Following incubation, explants were washed with PBS and eight explants were pooled and homogenized in ice cold RIPA lysis buffer (containing Complete Mini-EDTA free protease inhibitor and phosphatase inhibitors I and II) by using a glass/glass micro tissue grinder for 30 seconds. Tubes were then sonicated in an ultrasonic water bath for 15 seconds. After 30 minutes on ice, insoluble material was removed by centrifugation at 12,000×*g* at 4°C for 10 minutes and the supernatants were stored at −80°C until analysis. Protein concentrations were determined using the Bio-Rad Protein Assay with bovine serum albumin as a standard.

Protein samples (50 µg each) were separated on 4–20% polyacrylamide gels (Tris-HCl gel, Bio-Rad). After electrophoresis, the proteins were transferred onto nitrocellulose membranes (Pierce, Rockford, IL) that had been blocked with 5% non-fat dry milk in PBS with 0.1% Tween 20 (PBS-T). The membranes were incubated with primary antibodies, mouse anti-Prx3 (1∶1,200), anti-c-Myc (1∶1,000), or anti-GAPDH (1∶10,000) in 5% skim milk in PBS-T overnight at 4°C, and then washed three times (10 minutes each) with PBS-T. Secondary antibodies were applied at a concentration of 1∶10,000 for 1 hour at room temperature. Following extensive washing of the membrane, the immunoreactive bands were visualized with Enhanced Chemiluminescence Plus reagents.

X-ray films of Western blots were scanned and analyzed using AlphaEase software SpotDenso tool (Alpha Innotech, San Leandro, CA). The band densities were first normalized to background. Next, the probing protein/GAPDH ratio was calculated from the band densities run on the same gel. Finally, the difference in the ratio of the control and experimental bands was tested for statistical significance.

#### Quantitative RT-PCR

Explants were washed twice with PBS after incubation and kept in RNA*later*® until analysis. In order to obtain excellent RNA quality, total RNA was isolated from 10 pooled explants using the RNeasy Micro Kit due to the extremely minute samples that can be extracted from the explants. All tissues contributing to a pooled sample underwent the same treatment conditions. Quality and quantity of RNA were determined by RNA 6000 Pico Assay on a Model 2100 Bioanalyzer (Agilent Technologies, Palo Alto, CA) and Thermo Scientific Nano Drop™ Spectrophotometer (Thermo Scientific, Waltham, MA). RNA was stored at −80°C until analysis.

Total RNA (1 µg) was reverse transcribed to first-strand cDNA by using SuperScript III reverse transcriptase. The cDNA was diluted (1∶10) with DEPC-H_2_O and stored in aliquots at −20°C. Real-time PCR was performed in a Prism 7000 Sequence Detection System (Applied Biosystems, Foster City, CA) and triplicate reactions were run for each sample on a 96-well plate with the first-strand cDNA (template), Master Mix, and TaqMan probe for Prx3 (assay ID: Mm00545848_m1, Applied Biosystems). For normalization, S16-M49 (forward primer: TCAAAGGCCCTGGTAGCTTATTAC, reverse primer: CGATCGTATTGGATGAGGATATCTT) was also run on each plate as a standard. The *C*_T_ value (number of cycles at which the PCR reaction reaches an arbitrary threshold value) was calculated for each reaction [25].

#### Fluorescent assessment of hROS formation

After experimental treatment, the explants were washed twice with PBS. The media was exchanged for Dulbecco’s Modified Eagle Medium without phenol red and supplemented with 1% sodium pyruvate and 1% insulin-transferrin-selenium-A supplement containing 10 µM APF and 1 µM MitoTracker and incubated at 37°C and 5% CO_2_ for 30 minutes. The explants were then washed with PBS and placed on glass slides. Fluorescent images were immediately obtained on an Olympus Fluoview Confocal Laser Scanning Microscope-FV500.

#### Immunofluorescence assay for Prx3

Explants were fixed with 4% paraformaldehyde overnight at 4°C and then permeabilized with 3% Triton X-100 in PBS for 30 min at room temperature. The specimens were washed three times with PBS, blocked with 10% normal goat serum for 30 minutes at room temperature, and immunolabeled for Prx3 as described in the section “Immunofluorescence of Prx3 on surface preparations from adult mice” for *in-vivo* experiments in mice.

#### Quantification of fluorescent signals

The procedure for quantification of immunofluorescence of Prx3 or APF in organotypic cultures of explants was identical to that described in the above section “Quantification of immunofluorescence signals on surface preparations” for *in-vivo* experiments in mice.

#### Hair cell counts

Explants were fixed with 4% paraformaldehyde overnight at 4°C and then permeabilized for 30 minutes with 3% Triton X-100 in PBS at room temperature. The specimens were washed three times with PBS and incubated with Alexa Fluor 488 phalloidin (1∶200) or Alexa Fluor 594 phalloidin (1∶100) at room temperature for 30 minutes. After several rinses in PBS, the specimens were mounted on a slide with GEL/MOUNT™ (Biomeda Corp., Foster City, CA). The phalloidin-stained stereociliary bundles and circumferential F-actin rings on the cuticular plate outlining individual hair cells were viewed on a Leitz Orthoplan upright microscope equipped for epifluorescence, using a 50× oil-immersion objective. The right objective had a 0.17 mm calibrated scale imposed on the field for reference and the rows of inner and outer hair cells were oriented longitudinally within each 0.17 mm frame. Each successive 0.17 mm field was evaluated for missing IHCs and OHCs from the apex of the organ of Corti to the base. Cell counts were entered into a computer program (KHRI Cytocochleogram, version 3.0.6) for comparison with a normative database established from control specimens. The percentage of missing hair cells was calculated and plotted as a function of distance from the apex of the explant.

### Statistical Analysis

Data were statistically evaluated by one-way ANOVA with Student-Neuman-Keuls multiple comparison tests using Primer of Biostatistics software (McGraw-Hill Software, New York, NY). The statistical significance of the difference in relative fluorescence intensity or Western blot band density between treatment and control groups was tested with unpaired *t*-tests using GraphPad Software (GraphPad Software Inc., San Diego, CA).

## Results

### The Expression of Prx3 in Hair Cells is Affected by Inner Ear Insults in vivo

**Figure 1:**
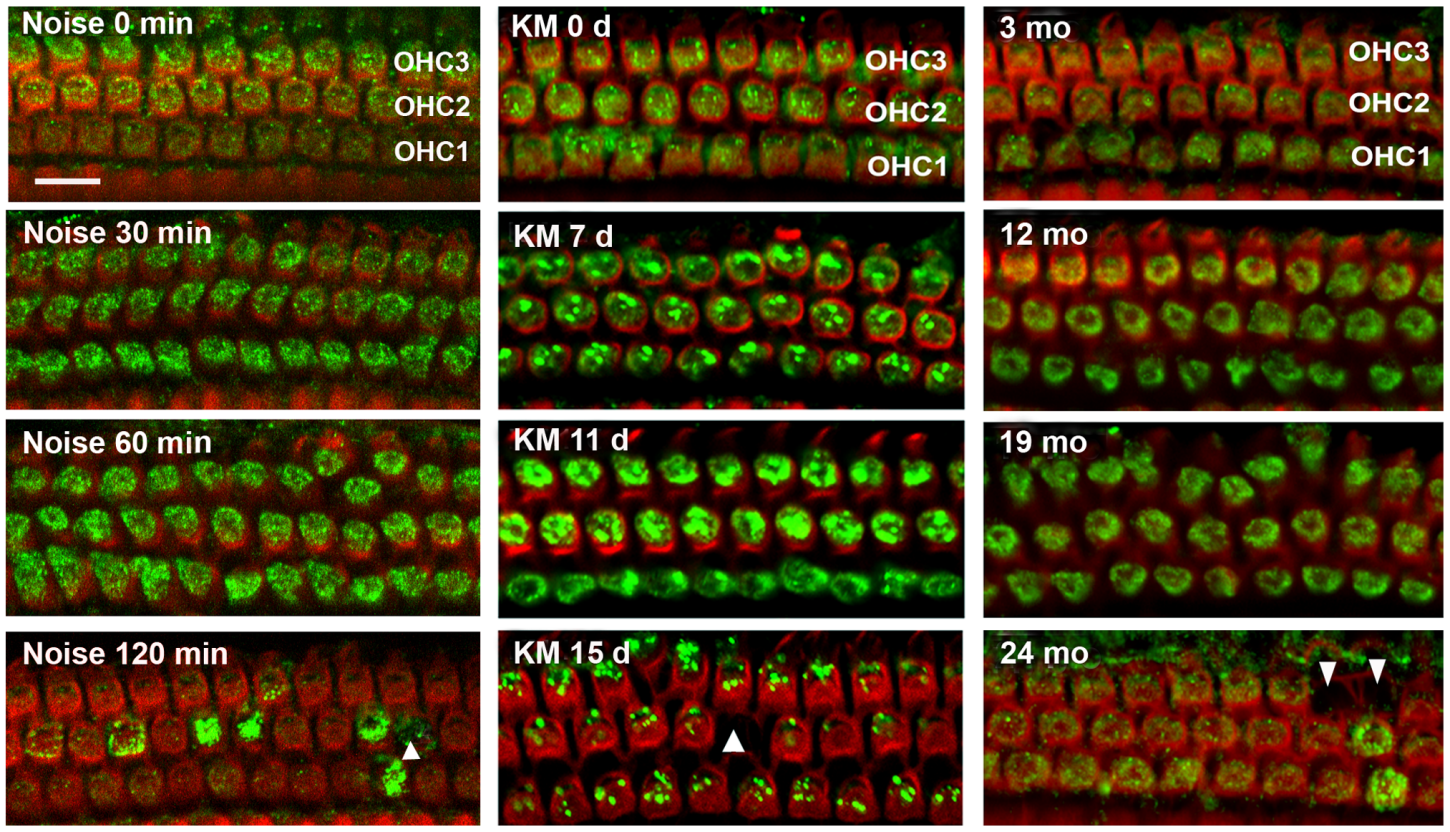
The levels of Prx3 in OHCs change in cochlear pathologies in vivo. Surface preparations from the upper basal turns of cochleae of CBA/J mice were labeled with a Prx3 antibody (green) and Alexa Fluor 594 phalloidin (red) after noise trauma, drug treatment, or aging. The left column illustrates the changes in Prx3-associated immunofluorescence with increasing duration of noise exposure (2–20 kHz BBN at 106 dB). The middle images show Prx3-associated immunofluorescence after increasing durations of kanamycin treatment. The right column of images demonstrates changes in Prx3-associated immunofluorescence signal with increasing ages. Quantification of Prx3-associated immunofluorescence revealed a significant increase after 30 to 60 minutes of the noise exposure, 7 to 11 days of kanamycin treatment, or 12 to 19 months of age (*p*<0.05, *n* = 3) respectively. ‘OHC1’, ‘2’, and ‘3’ indicate the first, second, and third rows of OHCs. Arrowheads indicate missing hair cells. Images are representative of three animals for each condition. Scale bar = 10 µm.

Although loss of auditory hair cells is a common feature of acquired hearing loss, the damage by noise, drugs, and age follow widely diverging time courses. The noise exposure used here, BBN from 2–20 kHz at 106 dB for 2 hours, is a relatively acute stress that results in hair cell loss in the basal region of the cochlear epithelium beginning 1 hour after noise [20]. Aminoglycoside ototoxicity develops over two to three weeks of daily injections with damage to hair cells progressing and stabilizing about a week thereafter [22]. Age-related sensory or neuronal hearing loss generally manifests in mammals past the middle of their lifespan and CBA/J mice follow such a pattern [21]. Despite the large variation in timing, the three insults had analogous effects on Prx3 levels *in vivo*. During the initial phase of increasing stress–after 30 to 60 min of noise exposure, 7 to 11 days of kanamycin injections, or 12 to 19 months of age–OHCs expressed increased Prx3 levels, while their structural integrity was maintained (fig. 1). Quantification of Prx3-associated immunofluorescence showed a two-fold, significant increase after 30 to 60 minutes of noise, 7 to 11 days of kanamycin treatment, or 12 to 19 months of age, respectively (*p*<0.05, *n* = 3). Furthermore, the increase in the band density of Prx3 protein during the aging process was confirmed by Western blot analysis using total cochlear homogenates (data not shown). Subsequently, Prx3 levels decreased drastically and hair cell loss began to occur. Alexa Fluor 594 phalloidin immunolabeling of the actin cytoskeleton showed scars on the cochlear epithelium from incipient loss of OHCs (fig. 1, arrowhead).

### Co-treatment with the Antioxidant DHB Increases Levels of Prx3 in OHCs and Prevents Kanamycin-induced Hair Cell Loss in vivo

**Figure 2:**
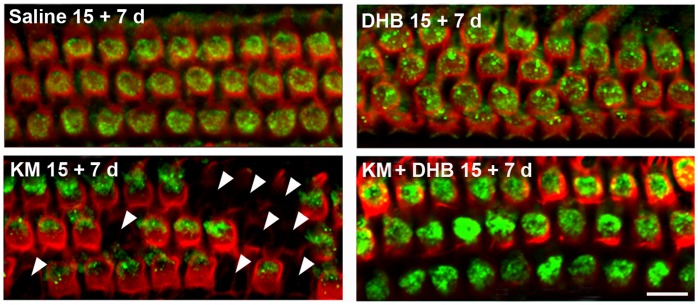
Co-treatment of kanamycin with the antioxidant DHB increases levels of Prx3 in OHCs and attenuates loss of OHCs in vivo. CBA/J mice were injected with saline, DHB, kanamycin, or were co-treated with kanamycin and DHB as described “in the methods section”. ‘15+7 d’ indicates a 15-day injection sequence followed by a 7-day rest period in order to observe maximum hair cell loss. Surface preparations of the upper basal turns of the cochlear epithelia of mice were then labeled with the Prx3 antibody (green) and Alexa Fluor 594 phalloidin (red) after kanamycin treatment. Quantification of the Prx3-associated immunofluorescence revealed a significant increase in OHCs by co-treatment of kanamycin with DHB (*p*<0.01, *n* = 3). Additionally, co-treatment of kanamycin with DHB significantly reduced hair cell loss (*p*<0.05, *n* = 3). Arrowheads indicate missing hair cells after kanamycin treatment. Images are representative of three animals for each condition. Scale bar = 10 µm.

Aminoglycoside-induced hair cell loss was used as a model to probe a correlation between antioxidant-mediated cell survival and Prx3 levels because the ability of DHB to attenuate kanamycin-induced hearing loss and hair cell death *in vivo* has been well characterized [26]. Concomitant administration of DHB with kanamycin resulted in stronger immunofluorescence for Prx3 than in kanamycin-only treatment (fig. 2) and quantitative analysis confirmed a two-fold increase (*p*<0.05, *n* = 3). Alexa Fluor 594 phalloidin immunolabeling of hair cells along the cochlear surface preparation from the base to apex showed little if any hair cell loss in mice co-treated with kanamycin and DHB, while considerable cell loss (arrowheads) was evident in the kanamycin-only treated mice.

### Delivery of Prx3 siRNA into the Ear Increases the Susceptibility to Noise-induced Hearing Loss

**Figure 3:**
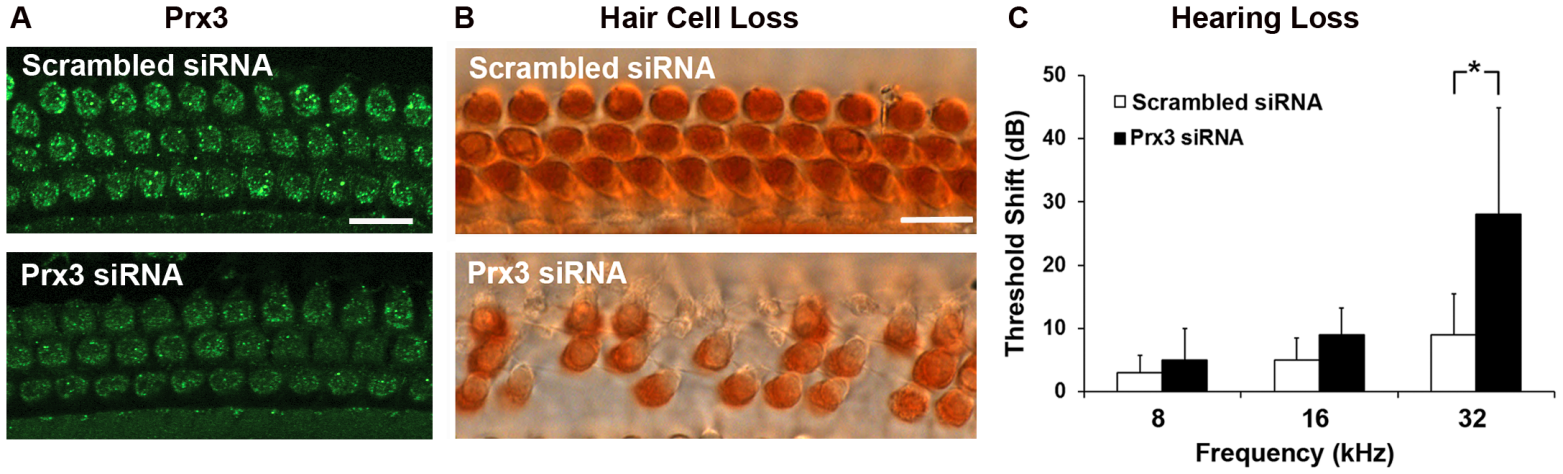
Prx3 siRNA reduces Prx3 expression in OHCs and increases hearing loss in vivo. A: Surface preparations of the upper basal turns of cochlear epithelia of CBA/J mice 3 days after Prx3 siRNA delivery were labeled with the Prx3 antibody (green). Images are representative of three individual animals. Scale bar = 10 µm. B: Hair cells outlined by myosin VII and DAB staining in the lower basal turn of the cochlea. Quantitative analysis of OHC loss in the lower basal turn 2 weeks after noise exposure (2–20 kHz BNN at 96 dB for 2 h) showed a significant difference between Prx3 siRNA and scrambled siRNA groups (*p*<0.05, *n* = 5). The images are representative of five mice analyzed for each condition. Scale bar = 10 µm. C: ABR threshold shifts. The bar graph represents the permanent hearing loss in 3-month old male CBA/J mice 2 weeks after noise exposure (2–20 kHz BBN at 96 dB SPL for 2 h). The threshold shift at 32 kHz was greater in the Prx3 siRNA group than the scrambled siRNA group. Data are presented as means+SD; *p<0.05, *n* = 5.

In a complementary experiment, the effect of interference with Prx3 expression by siRNA was tested in a noise trauma model. The noise model was chosen because its acute nature lends itself best for siRNA suppression of Prx3 expression during the course of an experiment. Using our recently developed intra-tympanic injection technique, 0.6 µg of Prx3 siRNA were delivered onto the round window in the mouse middle ear cavity [24]; the delivery suppressed the fluorescent signal of Prx3 in OHCs 3 days after siRNA delivery in comparison to scrambled siRNA controls (fig. 3A). Quantitative analysis confirmed that the level of Prx3 fluorescence in OHCs of siRNA-treated animals decreased in three of four animals and did not change in the fourth animal.

Three days after siRNA injections, mice were exposed to 96 dB BBN. The sound intensity was deliberately lower than in the initial studies of noise trauma (fig. 1) because we hypothesized that Prx3 suppression would sensitize the cochlea to noise exposure. Myosin VII and DAB staining of the cochlear epithelium showed increased loss of OHCs in the basal region of the cochlea in the Prx3 siRNA group (fig. 3B) and quantitative OHC counts confirmed a statistically significant difference in OHC survival between groups (*p*<0.05, *n* = 5). Consistent with loss of hair cells in the basal region of the cochlea, a high-frequency hearing loss was seen in the functional ABR measurements. Mice treated with Prx3 siRNA sustained an average threshold shift of 30 dB at 32 kHz, while control mice receiving scrambled siRNA prior to noise exposure maintained essentially normal hearing thresholds within 10 dB of baseline (fig. 3C; *p*<0.05, *n* = 5).

### Organ of Corti Explants Allow Detailed Study of the Regulation of Prx3

**Figure 4:**
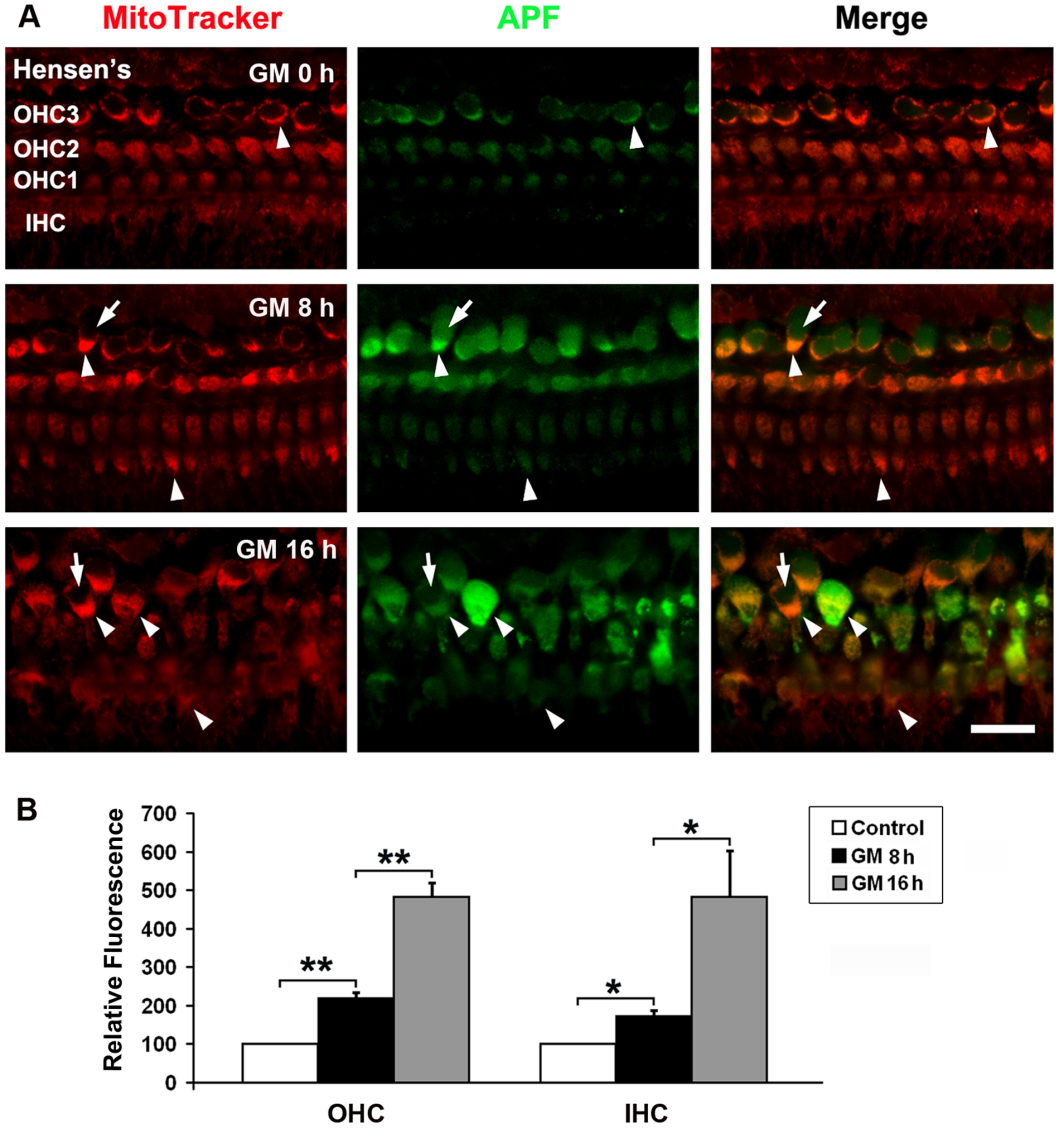
Gentamicin stimulates the formation of highly reactive oxygen species (hROS) in hair cells. A: Segments from the upper basal turn of organ of Corti explants were labeled with the hROS indicator APF (green) and with MitoTracker (red). ‘GM 8 h’ and ‘GM 16 h’ indicate the total hours of gentamicin (200 µM) treatment. ‘OHC1’, ‘2’, and ‘3’ indicate the first, second, and third rows of outer hair cells; ‘IHC’ indicates the row of inner hair cells. Arrowheads indicate APF fluorescence co-localized with mitochondria; arrows point to APF fluorescence localizing outside mitochondria. The images are representative of six preparations for each condition. Scale bar = 10 µm. B: The relative fluorescence intensities of APF with different lengths of gentamicin treatment were quantified for IHCs and OHCs as described “in the methods section”. Data are presented as means+SD; *p<0.05, **p<0.01, *n* = 6.

The mechanisms of Prx3 up-regulation in OHCs during the initial phase of insults were explored in explants of the mouse organ of Corti. This model, in which hair cells are sensitive to the addition of aminoglycosides in a time- and dose-dependent manner [23], lends itself more easily to direct manipulations. We used APF to detect highly reactive oxygen species (hROS) [27], [28] after incubation of explants with 200 µM gentamicin for 8 or 16 hours; these time points were chosen to precede the onset of significant hair cell loss. hROS fluorescence increased five-fold in hair cells of the gentamicin treated explants by 16 h compared to the control group (fig. 4A and B; *p<0.05, **p<0.01, *n* = 6). Furthermore, hROS fluorescence was mostly associated with mitochondria in hair cells, as indicated by co-localization with the mitochondrial marker MitoTracker, and only a small portion appeared to be cytoplasmic.

**Figure 5:**
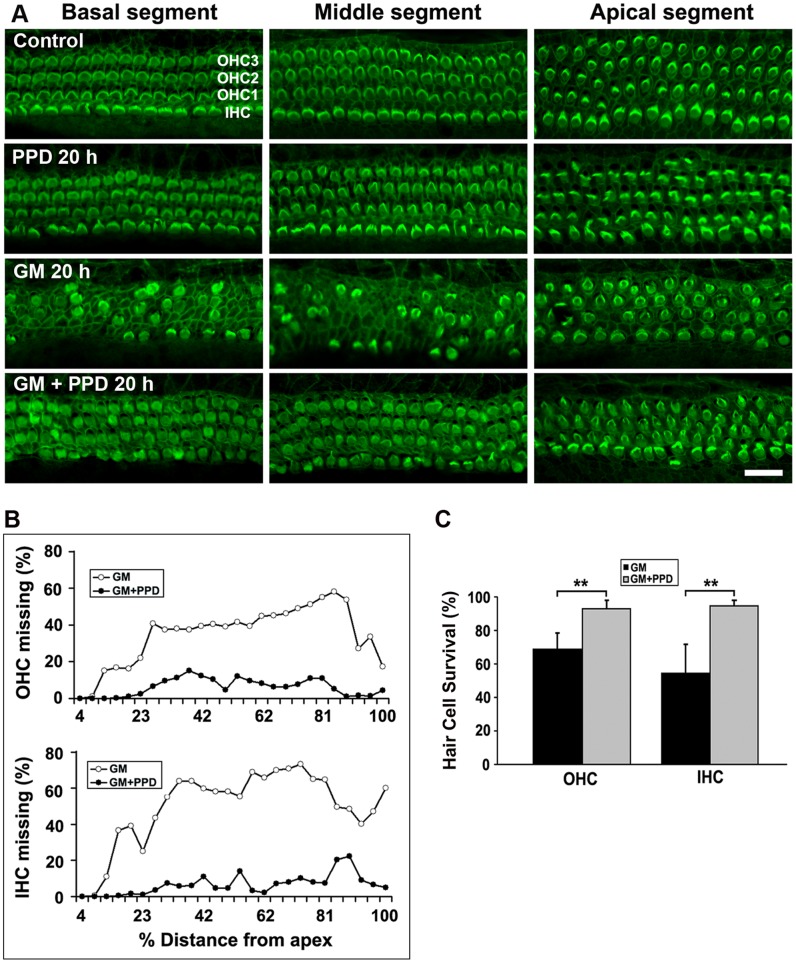
The antioxidant PPD prevents gentamicin-induced hair cell loss in organ of Corti explants. A: Organ of Corti explants were stained with Alexa Fluor 488 phalloidin to label outlines of sensory hair cells. ‘Control’ indicates incubation with 500 µM sodium sulfate for 20 hours; ‘PPD 20 h’: 1 mM PPD in culture medium for 20 hours; ‘GM 20 h’: explants treated with 200 µM gentamicin sulfate for 20 hours; ‘GM+PPD 20 h’: 200 µM gentamicin plus 1 mM PPD for 20 hours. The images are representative of seven preparations for each condition. Scale bar = 20 µm. B: Quantitative evaluation of loss of OHCs and IHCs. Cell loss was quantified along the entire cochlear spiral from the base to the apex of the explants after 20 hours of incubation with 200 µM gentamicin in the absence or presence of 1 mM PPD; *n* = 7 for each condition. C: Hair cell survival averaged over the length of the cochlea comparing treatment with 200 µM gentamicin alone and gentamicin in the presence of 1 mM PPD. Hair cell survival in the presence of PPD is significantly higher than for incubations with gentamicin alone. Data are presented as means+SD; **p<0.01* n* = 7. ‘IHC’ indicates inner hair cells; ‘OHC’ indicates outer hair cells.

Following the pattern of aminoglycoside-induced damage to auditory sensory epithelia in a wide variety of species, hair cell loss was most prominent in the basal and middle segments, while apical hair cells remained largely intact (fig. 5A). Consistent with ROS-mediated hair cell death, the free radical scavenger PPD (less prone to autoxidation *in vitro* than DHB) significantly protected hair cells from aminoglycoside damage, maintaining nearly normal morphology throughout the study (fig. 5). Quantitative hair cell counts confirmed the base-to-apex pattern of hair cell loss and the protection by PPD in all segments of the explant (fig. 5B and C; **p<0.01, *n* = 7).

**Figure 6:**
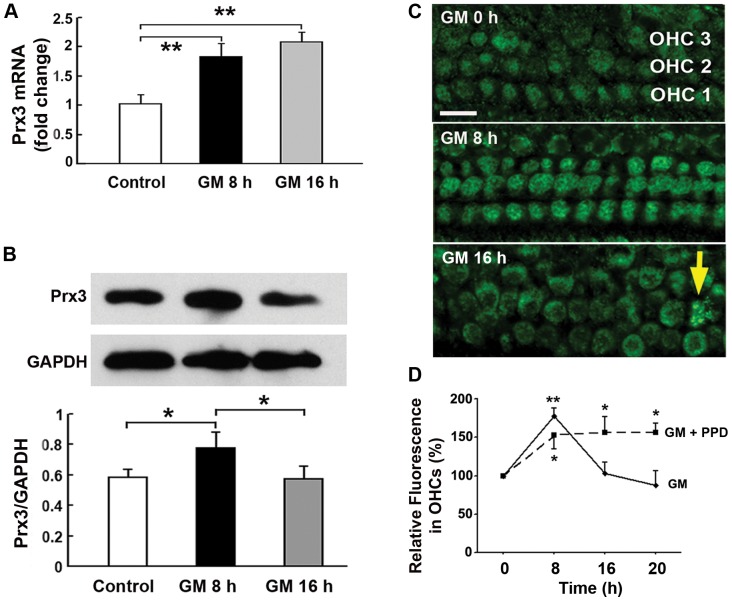
Gentamicin treatment changes mRNA and protein levels of Prx3 in the organ of Corti explants. A: Levels of Prx3 mRNA in explants were measured by qPCR analysis. ‘GM 8 h’ and ‘GM 16 h’ indicate the duration of treatment with 200 µM gentamicin; control incubations were carried out for 8 hours with 500 µM sodium sulfate as a substitute for gentamicin sulfate. Data are presented as means+SD; **p<0.01 for GM treatments vs. control, *n* = 5. B: Protein levels of Prx3 were detected in homogenates of pooled explants by Western blotting. Data are presented as means+SD; *p<0.05, *n* = 3. C: Representative basal segments from organ of Corti explants treated with 200 µM gentamicin and labeled with the Prx3 antibody (green) as used for quantification of relative fluorescence of Prx3 in OHCs. The arrow indicates Prx3 co-localized with a condensed nucleus. Scale bar = 10 µm. D: Quantitative analysis of relative fluorescence of Prx3 in OHCs after 8, 16, and 20 hours of treatment with gentamicin or gentamicin plus 1 mM PPD. Data are presented as means+SD, *p<0.05 vs. control, **p<0.01 vs. control, *n* = 6.

Finally, the amount of Prx3 mRNA in cochlear explants increased two-fold after aminoglycoside treatment (fig. 6A; **p<0.01, *n* = 5). In accordance with this increase and analogous to the *in-vivo* results, the levels of Prx3 protein were significantly elevated after gentamicin treatment by 8 hours (fig. 6B; *p<0.05, *n* = 3) and localized to OHCs. Eight hours is a time point when OHCs remained intact; Prx3 levels had begun to decrease by 16 hours when the OHCs began to die (fig. 6C). Co-treatment with the antioxidant PPD, which enhanced hair cell survival (fig. 5B, C), maintained elevated Prx3 levels from 8 to 20 hours (fig. 6D; *p<0.05, **p<0.01, *n* = 6 for each time point).

## Discussion

ROS are products and by-products of normal metabolism and contained within a physiological range by endogenous antioxidant systems. Peroxiredoxins are components of a ubiquitous thioredoxin-dependent antioxidant defense system. The specific localization of Prx3 in mitochondria, together with its mitochondrion-specific electron suppliers, namely thioredoxin 2 and thioredoxin reductase 2, provide a primary line of defense against superoxide and hydrogen peroxide produced by the mitochondrial respiratory chain. We should emphasize, however, that the focus on Prx3 in this study does not imply an exclusive role for this protein in containing ROS damage from auditory trauma. Rather, we consider Prx3 representative of more complex mitochondrial antioxidant systems.

There is good evidence that ROS formed in the inner ear following noise trauma, aminoglycoside treatment, and aging arise from mitochondria [29]. Excessive ROS production after these insults initially up-regulates antioxidant defenses but, upon prolonged exposure, oxidative damage occurs, affecting proteins, lipids, and DNA and triggering cell death pathways. In this study, we focus on the antioxidant Prx3 and evaluate its role in the determination of sensory hair cell death in diverse forms of acquired hearing loss. It is most intriguing that the response of Prx3 is so robust despite the diverging nature of the auditory insults: acute noise trauma causing damage within hours, drug-induced hair cell loss developing over several weeks, and age-related hearing loss emerging late in the lifespan of an animal. Furthermore, the levels of Prx3 correlate with cell survival, as seen in the biphasic time course of a transient increase during an initial challenge where hair cells remain intact and its decrease when cells are lost. Experimental manipulations of Prx3 levels further support this association.

The early elevation of Prx3 protein in hair cells in both the *in-vitro* and *in-vivo* studies is best understood as a homeostatic response, aimed to preserve the redox balance of the cells. This interpretation agrees with the evidence for ROS involvement in all three forms of auditory stress [9]–[11] and the established role of Prx3 in protecting cells from ROS-induced apoptosis by quenching intracellular and, in particular, intra-mitochondrial ROS [30]. Indeed, the current studies in mouse organ of Corti explants show that hROS arise in the mitochondria of hair cells during the early stages of gentamicin treatment and increase around the time when hair cell loss becomes evident. This demonstration supports the idea that aminoglycoside binding to mitochondrial RNA leads to mistranslation of proteins and mitochondrial dysfunction [15]. Likewise, ROS formation due to compromised mitochondrial function in noise trauma [17] and in the aging inner ear [31] would explain the Prx3 response in the other traumata. Continued stress and exhaustion of Prx3 will then ultimately lead to apoptotic (and, to a lesser extent, necrotic) death of sensory cells [32], [33]. While the role of Prx3 as an antioxidant is the most parsimonious explanation, Prx3 may additionally promote cell survival by enhancing LZK (leucine zipper-bearing kinase)-induced NF-κB activation [34]. This would be consistent with previous studies showing that the NF-κB pathway is essential for survival of OHCs from cochlear traumata [9], [35].

A more stringent and possibly causal relationship between Prx3 levels and cell fate is presented by the experimental manipulations to elevate or decrease Prx3. Radical scavengers have been well characterized to mitigate drug-induced and noise-induced ototoxicity by suppressing ROS formation in the cochlea [36], [37], but a connection with Prx3 levels is a novel concept. The additional presence of a radical scavenger during treatment with kanamycin *in vivo* maintains Prx3 levels and thereby protects against hair cell loss. The idea is strengthened by the results in organ explants where hair cells are also protected from aminoglycoside damage by an antioxidant. The complementary experiment using siRNA treatment to decrease Prx3 levels further underscores the protective role of Prx3. Under this condition, animals become more susceptible to noise trauma.

Finally, we should consider the apparent discrepancy between the time courses of changes in mRNA and protein levels in organ cultures challenged with gentamicin. At the 16-h time point, Prx3 mRNA remains elevated while Prx3 protein levels are decreased. We can speculate on two possible causes for this decrease. One is damage to mitochondria which could cause release of Prx3 and prevent mitochondrial targeting of newly synthesized Prx3; such Prx3 protein would be subject to degradation. Another possibility is that the prolonged gentamicin treatment inhibits cytosolic protein synthesis and reduces the rate (and efficacy) of translation of the RNA message into Prx3 protein [38].

In conclusion, Prx3 plays a crucial role in maintaining the homeostatic integrity of sensory hair cells and their defense against traumatic noise exposure, aminoglycoside challenges, and the aging process. The involvement of Prx3 in resistance or susceptibility to acquired hearing loss fits well with the notion that OHC damage is mediated by oxidative stress [12], [14], [18] and supports the role of Prx3 as an anti-apoptotic, homeostatic regulator that can be induced by oxidants [3].
